# Work-related stress is associated with low work ability, but not with poor self-rated health: A cross-sectional study in primary healthcare

**DOI:** 10.3233/WOR-230141

**Published:** 2024-08-06

**Authors:** Louise Danielsson, Anna Frantz, Kristina Holmgren

**Affiliations:** Department of Health and Rehabilitation, Institute of Neuroscience and Physiology, Sahlgrenska Academy, University of Gothenburg, Gothenburg, Sweden

**Keywords:** Occupational health, occupational stress, work performance, primary care, primary prevention, mental health

## Abstract

**BACKGROUND:**

People seeking care at primary healthcare centres may be exposed to work-related stress, increasing the risk of future sick leave. Thus, it is important to identify work-related stress, and to explore how stress relates to work ability and health.

**OBJECTIVE:**

To investigate the association between work-related stress and a) work ability, and b) self-rated health, among working women and men seeking care for physical or mental health complaints.

**METHODS:**

This cross-sectional study analyzed baseline data (*n* = 232) from a randomized controlled trial investigating the effects of a brief intervention to prevent sick leave. Data regarding work-related stress, work ability and self-rated health were analyzed using binary logistic regression models.

**RESULTS:**

In models adjusted for age, gender and education, high work-related stress measured by the Work Stress Questionnaire was significantly associated with low work ability. The highest odds ratio (OR 3.27, 95% CI 1.66–6.42) was found between the domain “interference between work and leisure time” and work ability, suggesting a more than three times higher odds for low work ability when perceiving that work interferes with leisure time. No significant association was found between work-related stress and self-rated health.

**CONCLUSION:**

Health professionals should explore patients’ work-related stress when they seek care for physical or mental complaints in primary healthcare. Patients’ perceived balance between work and leisure time seems particularly important to address. Increased awareness might facilitate timely, relevant strategies to reduce stress and promote work ability.

## 1 Introduction

During the past decades, stress on the job has increased across Europe [1]. A commonly faced problem in work life, work-related stress has broad negative consequences for the individual and their families, such as sickness and sickness absenteeism [2]. The reduced productivity and poorer performance in the organizations affect national economies negatively and, in turn, society at large. Consequently, work-related stress has turned into a major social phenomenon and urgent public health concern [3].

Work-related stress increases the risk of sickness absence [2, 4] and the risk of developing or worsening several health problems [5], for example musculoskeletal pain and common mental disorders. Common mental disorders (CMDs), including mild to moderate depression, anxiety disorders and stress-related illness, count for the majority of sick leave spells in Organisation for Economic Co-operation and Development (OECD) countries [6], often causing long sick leave periods and barriers in the return-to-work phase [7, 8]. Sick leave due to CMDs is increasing among young adults, risking labour-market marginalisation and a reduced workforce [9]. To increase knowledge and to shift this negative trend, it is essential to explore how work stress relates to work ability and health among workers seeking healthcare for physical or emotional distress.

Work ability can be defined as having occupational competence, health required for the competence, and occupational virtues that are required for managing the work tasks, assuming that the tasks are reasonable and that the work environment is acceptable [10]. A person’s performance at work depends on the complex interaction of personal, task-related, and organizational factors [11]. In a similar way, work-related stress connects both to organizational problems, such as indistinct work organization and leadership, increased workload, low influence and unresolved conflicts at work, and to high individual demands and commitments [4, 12, 13]. Work-related stress may also arise in the intersection between work and private life; women on sick-leave due to work-related stress describe the process of going from working to being sick-listed as losing control of work and private life, where personal and environmental factors together drive this process [14]. Interference between work and home, and vice versa, has been associated with a higher risk for sick-leave [15].

Previous research suggests that age and gender might influence work-related stress. Whereas cognitive irritation is more common in older workers, their higher emotional regulation skills may compensate to buffer work-related stress [16]. Regarding gender, women are more likely than men to experience mental health problems due to work-related stress [17]. Men and women have different exposures to stressors, and research from the COVID-19 period demonstrated increased stressors for women and mothers in terms of work-life conflict [18]. However, a recent meta-analysis showed no support for gender differences in the experience of work-related stress [19], suggesting that the type of occupation and gender balance at the workplace need further investigation as moderating factors. More consistently than gender, lower education has been associated with higher work-related stress [20].

Work-related stress links to mental and physical health problems in several ways. Depressive symptoms are more common at workplaces where psychosocial risk factors are present [21] and work-related psychosocial risk factors are associated with stress-related disorders [22]. A deterioration in mental health also increases the risk of co-morbid muskuloskeletal pain [23]. Workers experiencing work-related stress often seek care for musculoskeletal complaints (such as neck- or back pain) or psychological distress (such as sleeping disturbances) while still working, long before sick leave is even discussed [24].

In Sweden, primary healthcare provides first-line care for people with stress-related physical or psychological symptoms [8, 24], including medical and psychological treatments, referrals to specialized care when needed, and rehabilitation, such as physiotherapy and occupational therapy. Primary healthcare has the responsibility to assess the patient’s capacity to work, and, if needed, the general practitioner (GP) writes a sickness certificate suggesting full- or part time sick leave for a specific duration [25]. Given that primary healthcare is the first level of healthcare for people with stress-related problems, with a crucial role in the coordination of the patient’s subsequent care and the sick leave process, it is important to investigate work-related stress in this context. Since people seeking care in primary healthcare for musculoskeletal or psychological symptoms may be exposed to work-related stress, which increases the risk of future sick leave [4, 12], it is important that health professionals take notice of work-related stress in the patient’s narrative. Patients who report high work-related stress, have a two to four times higher odds of sick leave during the coming 12 months, compared to patients with low work-related stress [4]. This suggests that it is important to identify work-related stress early before the symptoms are severe and job functioning impaired. Possibly, the focus on work-related stress is overlooked in primary care, despite people seeking primary care for a vast range of symptoms linked to stress. Knowledge is still sparse on how work-related stress affects general health and perceived work ability in people seeking primary care for stress-related symptoms.

Primary healthcare encompasses the whole continuum of care, from health promotion and disease prevention to treatment and rehabilitation [26]. In recent years, governmental reports emphasize the need for more cohesive and collaborative healthcare, to increase health equity and prevent illness [27]. However, time for patient consultation is limited and there is a risk that important factors affecting health and work capacity might be overseen [25]. Increased knowledge about how work-related stress affects health and work ability could improve awareness among health professionals about when to discuss preventive measures or interventions with the patient to avoid future sick leave. While some research suggests that work-related stress is associated with reduced work ability in specific groups of workers [28, 29], there are, to the authors’ knowledge, no previous studies investigating this association in non-sick-listed people.

Thus, there is a need for more knowledge regarding work-related stress in non-sick listed people seeking primary healthcare for stress-related symptoms, and whether their work ability is affected. Moreover, little is known about whether perceptions of work-related stress relate to perceptions of general health in this patient group. To contribute to these knowledge gaps, the objective of this study was to investigate the association between work-related stress and a) self-rated work ability, and b) self-rated health, among working women and men seeking care for physical or mental health complaints in primary healthcare.

## 2 Methods

### 2.1 Study design

In this cross-sectional study, we analysed baseline data from the project Early identification of work-related stress (TIDAS). The project was carried out at primary healthcare centres in the Region Västra Götaland in Sweden between 2015–2017 and received the identification nr NCT02480855 in ClinicalTrials.gov [30]. The main aim of the TIDAS-project was to investigate whether a brief intervention, addressing work-related stress using the Work Stress Questionnaire (WSQ) [13] during a consultation with a GP, could influence sickness absence [30]. During the recruitment period, a research assistant was stationed at the healthcare centres to identify eligible recruits, collect informed consent, and administer questionnaires to the study participants. At inclusion, the participants filled out the WSQ together with information on demographics, self-rated health, and work ability. For the main study, data on sick leave was collected and analysed at 3, 6 and 12 months, which have been published elsewhere [31, 32].

### 2.2 Participants

Adults aged 18–64 years seeking care at primary healthcare centres, either for mental health issues or physical symptoms that could possibly be stress-related, were screened by the researcher stationed at the centre and, if eligible, invited to participate. Since stress relates to various symptoms, the inclusion was broad to capture both somatic manifestations such as musculoskeletal pain, headaches, gastrointestinal problems and dizziness, and mental complaints such as fatigue, sleeping problems, anxiety, or low mood. Other inclusion criteria were that the participants were employed or self-employed and currently working without sick leave or sickness benefit. They were not to have received sickness benefits at any point 1-month prior to inclusion. They were allowed to have ongoing medical, psychological and/or rehabilitation for their health problem. Exclusion criteria were any of following conditions: pregnancy, severe mental illness such as psychotic disorders, fractures, lump and spots, or an infection, or if the medical appointment was a planned check-up for a chronic disease, such as diabetes or chronic obstructive lung disease.

Eligible participants received verbal and written information about the study by the research assistant. They were able to ask questions about the study and were informed that they could withdraw at any time, and that declining or withdrawing from the study would not in any way affect their present or future healthcare. Those who wanted to take part in the study, signed a written informed consent form. The study was approved by the Regional Ethical Review Board.

### 2.3 Variables and assessments

Included participants were asked to fill in self-report measures of work-related stress, general health, and work ability, in connection to their clinical appointment. To assess work-related stress the Work Stress Questionnaire (WSQ) was used. WSQ is a self-administered questionnaire developed in a primary care context with the intention to identify individuals at risk of sick-leave [13]. The questionnaire consists of 21 questions covering both aspects of psychosocial working conditions and individual factors. The respondent is asked to rate the questions according to level of stressfulness on a 4-degree verbal ordinal scale. The questions are grouped into four domains: indistinct organisation and conflicts (7 items), individual demands and commitment (7 items), influence at work (4 items) and interference between work and leisure time (3 items). The median score for each domain can assume a value from 1 to 4 where 1 corresponds to not stressful and 4 corresponds to very stressful. The median score for each domain was calculated according to the instructions for the WSQ for each participant and used in the analysis. For the dichotomization, the cut off was set to 2 [4]. This was based on the qualitative phrasing and meaning of the items, where items representing score 1-2 are phrased with wordings “no” or “low” stress and 3-4 are phrased with wordings like “moderate” or “high” stress. This means that a median rating of 3-4 was interpreted as affirmed work-related stress for that domain. If an item could not be coded due to double ticking of boxes or if a participant missed an item, the participant was excluded from the analysis. The questionnaire has demonstrated satisfying face validity and acceptable test-retest reliability, with a 73% median percentage agreement of items among employed women, and 77% among men [13, 33].

To assess work ability the Work ability index (WAI) –single item was used. The respondents were asked to rate their current work ability on an ordinal scale from 0 to 10, where 0 corresponds to no work ability and 10 corresponds to the participant rating work ability to be at its best. WAI is commonly used to assess work ability in both research and clinical practice [34]. WAI single item was developed as a brief, valid assessment to efficiently measure work ability and has shown a strong association (Spearman’s coefficient 0.87) with the full instrument among Swedish women on sick leave [35]. Convergent validity and similar results between the single item and the full WAI have been demonstrated in employed workers [36].

Self-rated health was measured using the first, general health question from the Swedish version of Short Form Health Survey-36 (SF-36). The respondents were asked to rate their general health on a 5-degree ordinal scale. Possible responses were excellent, very good, good, fair, and poor corresponding to the numbers 1–5, where 1 is excellent and 5 is poor. The Swedish version of SF-36 has demonstrated good reliability and construct validity in the general population [37].

### 2.4 Data analysis

Only participants with complete responses in the WSQ questionnaires were included in the present study. If the participant had missed an item in a domain, or if there was a double ticking of boxes, the participant was excluded from analysis. Thus, no statistical strategy to manage missing data, such as imputation, was used.

Data was analysed using SPSS statistics v. 27 (IBM Corp., Armonk, NY, USA). Descriptive statistics were used to describe the characteristics of the study participants and their reported work-related stress, work ability and self-rated health. Binary logistic regression was used to analyse the relationship between a) work-related stress and work ability, and b) work-related stress and self-rated health. In line with previous studies using the WSQ in logistic regressions [4, 12, 38] we chose to dichotomize the four categorical responses of the variable into a binary outcome of low or high stress. We assumed that this definition would be of higher clinical value for identifying patients at risk, rather than differentiating the level of stress by the four categories. Thus, work-related stress was dichotomised using the median for each domain, where 1-2 was defined “no/low stress” and 3-4 was defined “high stress”. Work ability was dichotomised using the median rating (7) for this variable. Work ability rated 0 to 6 was defined “poor” and 7 to 10 was defined “good”, in line with the suggested cut off 7 [39]. Self-rated health was dichotomised as “good” (categories 1–3) or “poor” (categories 4-5). The qualitative meaning of the response categories was used to determine the cut-off, where categories 1 through 3 have a positive connotation to health and 4 through 5 have a negative. The models were adjusted for gender, age, and educational level, which we assumed would be factors influencing work stress, based on previous studies [16, 18, 20]. To allow for assessment of each variable’s effect on the model, a stepwise approach was used. First, the regression was performed without any adjustments. In the second model, gender was added as a binary covariate. In the third model, age (categorized into age groups), was added to the second model. In the fourth model educational level (categorized) was added to the third model.

To examine multicollinearity among the independent variables, correlation coefficients and variance inflation factor (VIF) were calculated. There is no established cut-off for multicollinearity, however a correlation coefficient above 0.8 indicates a serious multicollinearity problem [40]. For VIF, a number below 2.5 has been suggested to indicate low risk for multicollinearity in logistic regression models [40]. Therefore, VIF should be smaller than 2.5 to be acceptable.

## 3 Results

A total of 271 participants were recruited for the RCT study. Of these, 232 had complete answers to the WSQ and were included in the analysis. There was a 99% response rate to the question about work ability (2 missing) and 95% response rate to the question about perceived health. The characteristics of the study sample are presented in Table 1. A major proportion of the participants identified as female (66%) and cohabiting with a partner (74%). 44% had received education at university level and half of the study sample were in the ages 31–50. Mean age was 43.8 years, with a standard deviation of 11.7. The most common reasons for seeking care were related to mental health issues such as depressed mood, insomnia, or stress or pain syndromes such as pains in joints, muscles, or headaches. Overall, the general self-rated health among the participants was good (73%). For work ability, almost half of the participants (45%) rated their work ability as low.

**Table 1:** Characteristics of the study participants

	Number of	Proportion
	participants	(%)
	Total (*n* = 232)
	(*n* = 232)
Gender
Male	79	34
Female	153	66
Age (years)
18–30	41	18
31–50	117	50
51–64	74	32
Educational level
Elementary or high school	128	55
University or higher	103	44
education
Missing data	1	1
Occupational class
Skilled/unskilled manual	89	38
Medium/low non-manual	100	43
High-level non-manual	42	18
Missing data	1	1
Marital status
Single	45	19
Married/cohabitant	172	74
In a relationship, living separately	13	6
Missing data	2	1
Reason for seeking healthcare
Musculoskeletal problems or headache	69	30
Mental health problems	83	36
Cardiovascular problems	20	8
Gastrointestinal problems	30	13
Other (i.e dizziness)	11	5
Non-specified (i.e “feeling unwell”)	8	3
Missing data	11	5
Self-rated work ability
High	127	55
Low	103	44
Missing data	2	1
Self-rated health
Good	168	73
Poor	52	22
Missing data	12	5

**Fig. 1:**
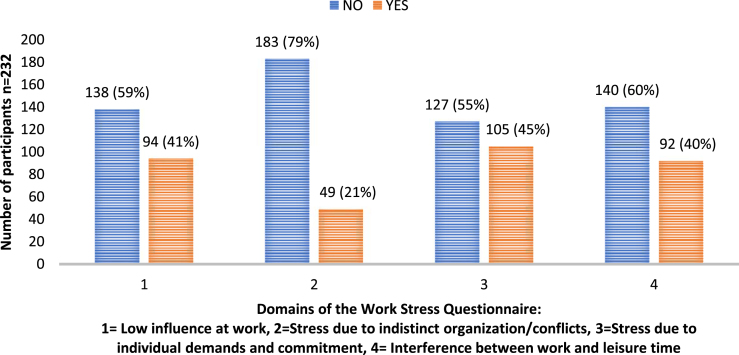
Perceived work-related stress among the study participants, measured using the Work Stress Questionnaire and dichotomised as “no” (median value of 1–2) or “yes” (median value of 3—4) for the respective domains. Total *n* = 232.

The ratings of work-related stress among the study participants are presented in Fig. 1. There was a higher reported rate of perceived stress related to high individual demands/commitment, low influence at work and imbalance between work and leisure time (40–45%) than the reported rate of perceived stress related to indistinct organisation and conflicts (21%).

The logistic regression performed to examine the association between work-related stress and work ability showed that the model was statistically significant (chi square = 55.65, df = 4, *p* < 0.001). The model explained 29 % (Nagelkerke R^2^) of the variance in work ability and correctly classified 70% of cases. High work-related stress in three of the four domains was significantly associated with low work ability, results are presented in Table 2. The highest ORs for low work ability was found in the significant associations (*p* < 0.01) with high stress related to interference between work or leisure time (OR 2.69, 95% CI 1.44–5.01) and with low influence at work (OR 2.60, 95% CI 1.40–4.81). Adjusting for age and gender, respectively, did not change the association significantly, but the adjusted model displayed slightly increased ORs compared to the unadjusted model. When adjusting for educational level in model 4, all four domains of the WSQ demonstrated that high stress was significantly associated with low work ability (*p* < 0.05). Lower educational level increased the odds ratio in the association between low work ability and high work stress with 0.2–0.5 times for the different work-related stress domains.

**Table 2:** Unadjusted and adjusted odds ratio (OR) with 95% confidence interval (CI) for the association of low work ability when exposed to high work-related stress, reported for each domain of the Work Stress Questionnaire

	Model 1 OR (95% CI)	Model 2 OR (95% CI)	Model 3 OR (95% CI)	Model 4 OR (95% CI)
Low influence at work	2.602^**^ (1.399–4.838)	2.894^**^ (1.535–5.459)	2.900^**^ (1.536–5.474)	2.957^**^ (1.469–5.955)
High stress due to indistinct organisation and conflicts	1.892 (0.893–4.008)	1.848 (0.860–3.967)	1.820 (0.843–3.930)	2.438^*^ (1.075–5.528)
High stress due to individual demands and commitment	2.182^*^ (1.164–4.092)	2.367^*^ (1.249–4.483)	2.366^*^ (1.249–4.484)	2.812^**^ (1.393–5.677)
Interference between work and leisure time	2.687^**^ (1.436–5.028)	2.849^**^ (1.510–5.375)	2.875^**^ (1.519–5.439)	3.265^**^ (1.660–6.421)

Model 1 unadjusted OR. Model 2 adjusted OR for gender. Model 3 adjusted OR for Model 2 with the addition of age. Model 4 adjusted OR for Model 3 with the addition of educational level. ^*^p < 0.05 ^**^p < 0.01.

None of the four domains of work-related stress (influence at work, high risk due to indistinct organisation and conflicts, high stress due to individual demands and commitment, and interference between work and leisure time) was significantly associated with poor self-rated health (Table 3). Adjusting for gender, age and educational level did not change the model significantly.

**Table 3:** Unadjusted and adjusted odds ratio (OR) with 95% confidence interval (CI) for the association between self-rated poor health and high work-related stress for each domain of the Work Stress Questionnaire

	Model 1 OR (95% CI)	Model 2 OR (95% CI)	Model 3 OR (95% CI)	Model 4 OR (95% CI)
Low influence at work	1.845 (0.929–3.665)	1.875 (0.938–3.749)	1.900 (0.947–3.812)	1.884 (0.937–3.790)
High stress due to indistinct organisation and conflicts	0.802 (0.361–1.781)	0.794 (0.357–1.768)	0.860 (0.382–1.939)	0.878 (0.387–1.991)
High stress due to individual demands and commitment	1.558 (0.767–3.165)	1.574 (0.772–3.206)	1.597 (0.783–3.254)	1.731 (0.835–3.585)
Interference between work and leisure time	1.555 (0.779–3.103)	1.565 (0.784–3.128)	1.496 (0.744–3.007)	1.521 (0.753–3.069)

Model 1 unadjusted OR. Model 2 adjusted OR for gender. Model 3 adjusted OR for Model 2 with the addition of age. Model 4 adjusted OR for Model 3 with the addition of educational level. ^*^p < 0.05 ^**^p < 0.01.

The independent variables in the models were analysed for multicollinearity, displaying the highest correlation coefficient between gender and educational level (*r* = 0.188). The VIF ranged from 1.051 to 1.175, indicating low risk for multicollinearity among the independent variables.

## 4 Discussion

### 4.1 Discussion of the results

The key finding of this study was that high work-related stress was associated with low work ability, but not with self-rated general health, in patients seeking primary healthcare. We found that the odds for low work ability were more than two times higher in patients reporting high work-related stress (related to high individual demands and commitment, low influence at work or interference between work and leisure time), compared to those reporting no or low work-related stress. The odds increased when adjusting for gender, age, and educational level, and in the adjusted model high stress due to indistinct organisation and conflicts was also associated with low work ability. Taken together, our findings indicate that not only is poor organizational climate associated with low work ability [29, 41], but also personal aspects of work-related stress, such as high individual demands and commitments, and interference between work and leisure time, seem to be associated with low work ability in adults seeking primary healthcare. While the organizational climate may be difficult to influence from the perspective of healthcare, it has been shown that early identification of work-related stress in primary care affected the patient’s treatment plan, including timely and tailored psychological and pharmacological treatments [32, 42]. Thus, exploring together with the patient about their views on work demands and commitment, and how to balance work and leisure time, should be relevant within the scope and responsibilities of primary care. Collaboration and teamwork between different healthcare professionals could improve such health-at-work promotion. For example, work-related stress can be identified by a GP during the clinical appointment [32] and suggested strategies for recovery or adjustments might be provided by psychologists, physical or occupational therapists.

We did not find an association between work-related stress and self-reported health, measured by the single question from SF-36. This was somewhat unexpected, since previous research have suggested that work-related stress negatively affects health outcomes in the general working population [38, 43]. However, the SF-36 health concept have demonstrated better sensitivity to physical health complaints than to mental distress [44], which was commonly experienced in our sample. Possibly, the generic health question from SF-36 did not capture early, milder signs of perceived mental distress. It has been suggested that interpretation of the item varies and that some respondents may refer to specific health problems while others consider their overall functioning [45]. Moreover, critique has been raised towards using only the general question of the SF-36, arguing that this question cannot be regarded a composite for the different domains of the scale [46]. It is also possible that the single health question was interpreted by the respondents in our study as referring to their overall physical health rather than symptoms of stress. Another generic patient-reported outcome measure might have better identified health issues related to mental distress, for example the General Health Questionnaire-12 [47].

In this study, lower education increased the association between work-related stress and low work ability. Possibly, workers with higher education have different coping strategies and more flexibility in their job tasks, to sustain their work ability in the presence of stress [48]. In line with our results, a Swedish population study demonstrated higher sick leave incidence among people with 9–10 years education compared to people with higher education [49]. Interestingly, another study found a higher risk of poor quality of life among highly educated employees experiencing work stress [50]. The authors discuss the possibility of higher self-expectations on work ability, resulting in a gap between expectations and work status, which could impact negatively on quality of life. Further studies are warranted to clarify the role of education level in workers’ perceptions of work-related stress.

Our results suggest that gender and age do not significantly affect the association between work-related stress and work ability, or between work-related stress and health perception. While women are more commonly sick-listed due to stress-related disorders compared to men [51], work ability is affected in a similar way across gender in female-dominated work sectors, where a similar type and level of work stress can be expected [52]. Previous research has reported similarities between women and men in their response to stress, suggesting that other factors related to the labour market, organisation or gender inequality are more likely to impact work ability [52]. In gender-equal countries, depression levels are more similar between women and men than in countries with higher gender inequality, especially in people of younger age [53].

While the data for this study was collected before the Covid-19 pandemic, the findings are relevant also for the working population in the post-pandemic phase. A meta-analysis of studies investigating the impact on mental health found a higher prevalence of depression, anxiety and psychological distress compared to before the pandemic [54]. The need for addressing mental health issues from a work perspective has likely increased during this period.

Since low work ability has been associated with future sick-leave [55], addressing work stress as a factor contributing to reduced work ability is important both for the individual, their families, and society. A brief intervention where GPs reflected together with the patient about their perceived work-related stress did not have an impact on future sick-leave [31], which suggests a need for a more in-depth or collaborative approach between the patient, healthcare and workplace. In this study, perceived interference between work and leisure time displayed the highest odds for low work ability, suggesting that the patient’s work-life balance and recovery time are particularly important to explore and adjust.

### 4.2 Methodological considerations

A strength of this study was the naturalistic approach of including working adults seeking primary care for both mental and physical complaints, thereby acknowledging the complexity of signs of work-related stress. Another strength, which provides additional knowledge compared to register-based studies, was the use of patient-reported outcome measures. Also, since the Work Stress Questionnaire captures and differentiates between different stress domains, it enabled a more nuanced understanding of the investigated associations.

While the WSQ has four possible responses for each domain, from not stressful to very stressful, the main clinical task is often to differentiate between low and high stress to identify individuals at risk. Previous studies investigating associations between work-related stress, measured by the WSQ, and several health or functional outcomes, have dichotomized the four WSQ response alternatives into low/high stress [4, 12, 38]. A similar procedure was used for the present study, which also increased the number of observations for each alternative (low/high). However, it is important to note that information may get lost when dichotomizing a categorical variable, possibly hampering the interpretation of the logistic regression [56].

While the WAI single item has been suggested as an acceptable brief alternative to the full scale, in both working [36] and sick-listed populations [35], a recent study found that construct validity was insufficient in a sample of patients with low back pain, the majority being employed and working [57]. Thus, another measure to capture work ability could have been preferable in our similar sample. After the data for our study was collected, it has been suggested that three of the WAI items, rather than the single item, may be suitable proxies for the full scale [58].

A weakness of the study is that causality in the analysed correlations cannot be assumed in a cross-sectional design [59]. Also, the assumption of linearity in logistic regression models may be problematic in cases of complex relationships between variables, typically with continuous independent variables. However, since our models were performed with binary or categorical variables only, the issue of linearity should be less pertinant. While there are other, more complex statistical alternatives, logistic regression has advantages in terms of being well known, and easy to perform and interpret [60].

Another limitation is that we lacked data on the participants’ work-related stress prior to inclusion, which could have given important information about the influence of the duration of stress on work ability and health.

Only 21% of the participants reported high stress owing to indistinct organisation and conflicts among the study participants, resulting in a small subgroup in the analysis. This may have affected the possibility to reach significance for this domain in relation to work ability and health. A larger sample could have made it possible to detect a small but significant association.

Some factors affect the generalizability of the results. First, there was a high representation of women among the study participants. It was only possible to choose between male or female gender in the questionnaire, and thus the results cannot be generalized to other gender identities. Second, there was low representation of participants born in other countries than Sweden and no other data on ethnic or cultural background were collected. This would have provided important information, since previous research has identified differences in sick leave patterns between native Swedes and migrated Swedes, suggesting an all-cause higher risk for sick leave among migrated Swedes but a higher risk for sick leave due to mental disorders among native Swedes [49]. Also, the Swedish primary care context limits the generalizability to countries with a similarly structured healthcare. However, work-related stress is a global threat to health and productivity [61], supported by a recent post-pandemic survey [62], where a third of the respondents reported at least one health problem (fatigue, headaches, muscle problems or pain) caused or made worse by work. Thus, knowledge about different dimensions of work-related stress, as measured by the WSQ, and how it links to work ability and health should be useful across countries, regardless of primary healthcare structure. Particularly, we believe that the association between imbalance between work-leisure time and poor work ability may be transferable. Noteworthy is also that the health professionals themselves may be at risk developing work-related stress [63]. Increased knowledge might also improve self-awareness among healthcare providers to enable preventative measures.

## 5 Conclusion

Work-related stress among working adults seeking primary healthcare for mental or physical health-complaints was associated with low perceived work ability. While this association should be interpreted with caution, the findings indicate that patients with various symptoms may experience work-related stress and decreased work ability, although these are not their primary reasons for seeking healthcare. This suggests a need for health professionals to explore the patient’s job situation, and to discuss relevant strategies to prevent increased symptoms and future sick leave. Based on the results, it is important to address the balance between the patient’s work and leisure time, including ways to increase recovery, or other adjustments to relieve stress. Given the complexity of health at work, where also job tasks, management and the workplace play important parts, we suggest that future research in primary care could focus on ways to effectively promote and communicate about work-life balance and recovery activities. Qualitative studies could explore successful examples and strategies from the perspective of patients and health professionals.

## Ethical approval

Ethical approval for the RCT was obtained by the Ethical Review Board in Gothenburg, no. 125-15. An additional application for the specific aim of the present study was approved by the Swedish Ethics Review Authority, no. 2021-00627.

## Informed consent

Written informed consent was obtained from the study participants upon inclusion to the RCT study stating the voluntary nature of participation in the study and the possibility to withdraw at any time.

## Conflict of interest

The authors declare that they have no conflict of interest.
